# Elderly male smokers with right lung tumors are viable candidates for *KRAS* mutation screening

**DOI:** 10.1038/srep18566

**Published:** 2016-01-07

**Authors:** Yang Yang, Chun Shi, Hui Sun, Wei Yin, Xiao Zhou, Lei Zhang, Gening Jiang

**Affiliations:** 1 Department of Thoracic Surgery, Shanghai Pulmonary Hospital affiliated Tongji University, Shanghai 200433, China; 2 Department of Oncology, Shanghai Pulmonary Hospital affiliated Tongji University, Shanghai 200433, China; 3 State Key Laboratory of Virology, College of Life Sciences, Wuhan University, Wuhan 430072, China; 4 The State Key Laboratory Breeding Base of Basic Science of Stomatology (Hubei-MOST) & Key Laboratory of Oral Biomedicine Ministry of Education, School & Hospital of Stomatology, Wuhan University, Wuhan, 430079, China; 5 Department of Endodontics & Periodontics, College of Stomatology, Dalian Medical University, Dalian 116044, China; 6 Lung Cancer Diagnosis and Treatment Center, Shanghai Pulmonary Hospital affiliated Tongji University, Shanghai, 200433, China

## Abstract

Genetic aberrations in tumor driver genes provide specific molecular targets for therapeutic intervention, which can greatly improve therapeutic outcomes. Here, we analyzed the mutational frequency of *EGFR* and *KRAS* gene, as well as *EML4-ALK* rearrangement, and summarized the clinicopathological characters of Chinese lung cancer patients. We detected the mutation spectrum of 1033 primary lung cancer patients. The analyzed clinicopathological parameters included gender, age at diagnosis, smoking status, pathological TNM stage, tumor morphology and location, visceral pleural invasion, and histological type. A total of 618 patients had mutations in *EGFR* or *KRAS* gene as well as rearrangement of *EML4-ALK*. Exon 19 deletions and L858R in the *EGFR* gene were the most frequent mutations. Left-side lung cancer was more common in female patients carrying the *KRAS* mutation. Rearrangement of *EML4-ALK* was more common in non-tobacco-using male patients, who also exhibited a higher likelihood of visceral pleura invasion. Elderly females who never smoked and possessed 1–20 mm stage I adenocarcinomas in the right side exhibited a higher frequency of *EGFR* mutations. Elderly male smokers with right lung tumors were viable candidates for *KRAS* mutation screening.

The global cancer burden is growing at an alarming rate, emphasizing the need for the urgent implementation of effective prevention strategies. Lung cancer is one of the most critical types, accounting for 13% of cancer diagnoses in 2012. The 5-year relative survival rate of lung cancer patients is gradually improving due to improvements in treatment. However, lung cancer remains the most common cause of cancer death, with 1.6 million patient deaths worldwide in 2012. In China, lung cancer also ranks highest in both cancer prevalence and lethality^1^. According to histological analyses, lung cancer is classified into non-small cell lung carcinoma (NSCLC), which consists of three main subtypes (adenocarcinoma, squamous cell carcinoma, and large cell carcinoma), and small cell lung carcinoma. Rare subtypes include glandular tumors, carcinoid tumors, and undifferentiated carcinomas.

Recently, a growing number of oncogenic mutations have been identified in lung cancer. Molecular genetic analyses have suggested that anaplastic lymphoma receptor tyrosine kinase (*ALK*), v-akt murine thymoma viral oncogene homolog 1 (*AKT1*), B-Raf proto-oncogene, serine/threonine kinase (*BRAF*), epidermal growth factor receptor (*EGFR*), v-erb-b2 avian erythroblastic leukemia viral oncogene homolog 2 (*ERBB2*), Kirsten rat sarcoma viral oncogene homolog (*KRAS*) and phosphatidylinositol-4,5-bisphosphate 3-kinase, catalytic subunit alpha (*PIK3CA*) are driver genes in NSCLC^2^,^3^,^4^,^5^,^6^. These genetic aberrations provide specific molecular targets for therapeutic intervention, which can greatly improve therapeutic outcomes. Erlotinib and gefitinib, EGFR tyrosine kinase inhibitors (TKIs), and crizotinib, an ALK inhibitor, have generated significant improvements in the objective response rate and resulted in longer progression-free survival^7^,^8^.

However, these specific inhibitors only function effectively when used in the correct population. Thus, accurate prevalence, clinical, pathological, and mutational status data are required. In this study, we extended the effort to analyze the phenotype-genotype relationship in Chinese lung cancer patients. We hypothesized that the clinicopathological parameters, including the age, gender, tumor size and site, TNM stage and genotype, of different patients groups are distinct. Based on these results, clinicians can adopt these principles in selecting treatment plans.

## Materials and Methods

### Ethical approval

This study was conducted in accordance with the amended Declaration of Helsinki. This study was approved by the Institutional Review Board (IRB) of Shanghai Pulmonary Hospital affiliated with Tongji University (20120163). Written informed consent was obtained from all participants. The methods were carried out in accordance with the approved guidelines.

### Patients and specimen collection

A total of 1033 consecutive primary lung cancer patients who were admitted into the Shanghai Pulmonary Hospital affiliated with Tongji University from January 2013 to January 2014 were recruited. No choose or correct was performed on patients collection. None of these patients received any anticancer therapies prior to surgery. The recurrent or metastatic patients were excluded. All of the cancer cases were confirmed by pathological tests or/and radical surgical resection. Fresh primary tumor tissues that contained more than 50% tumor cells were collected during surgery.

The clinical and pathological data obtained for analysis included gender, age at diagnosis, smoking status, pathological TNM stage, tumor morphology and location, visceral pleural invasion and histological type. The clinical information was recorded three times by three physicians respectively to ensure the accuracy. Tumors were staged pathologically according to the Union for International Cancer Control (UICC-7) staging system for lung cancer^9^.

### Candidate gene mutation analysis

Genomic DNA and total RNA were extracted from fresh tissues using the QIAamp DNA Tissue Kit and RNeasy Kit (Qiagen, Germany), respectively. Mutations in the *EGFR* and *KRAS* genes, as well as *EML4-ALK* rearrangement, were detected using Amoy Diagnostics Kits (Xiamen, China). The kits employ distinctive real-time PCR technology to detect mutations in the target gene. Target DNA is amplified with mutation-specific PCR primers, and the mutant amplicons are detected with a novel fluorescent probe. The test can detect mutations at a sensitivity of 1%. The positive and negative controls were provided by the manufacturer.

### Statistical analysis

Χ^2^ tests were performed to analyze the association between the gene variants and other clinicopathological data. All data were analyzed using the SPSS package for Windows (Version 18.0, Chicago, IL). *P* values <0.05 was considered statistically significant.

## Results

### Clinicopathological characteristics

**Figure 1:**
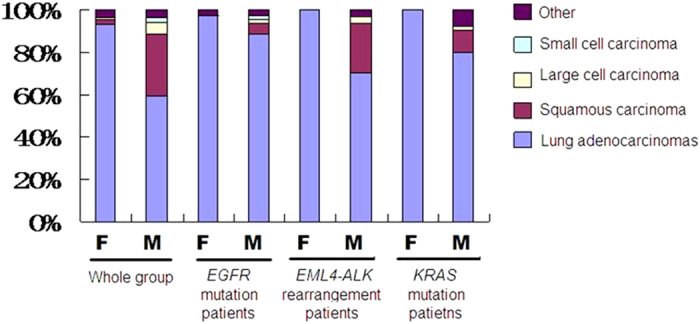
The pathologic profiles of the whole population and different mutation subgroups.

A total of 1033 lung cancer patients were recruited. More than 99% of them are Han people. The mean age of them was 60.68 years old (ranging from 24 to 91). Of these patients, 597 (57.79%) were male, and 624 patients, most of whom were female, had never smoked. 414 (40.08%) and 592 (57.31%) of tumors were located in the left and right sides, respectively. Visceral pleural invasion was observed in 335 patients. Histologically, 759 (73.48%) specimens were lung adenocarcinomas, 189 (18.30%) were squamous carcinoma, 32 (3.10%) were large cell carcinoma, and 15 (1.45%) were small cell carcinoma (Table 1; Fig. 1).

### Mutation spectrum

**Figure 2:**
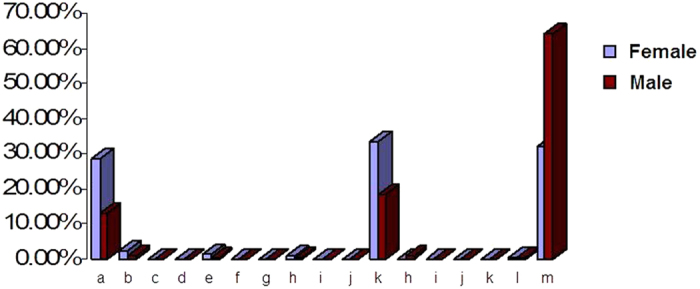
The *EGFR* mutations identified in this study. a–m: 19 del; 19-del/L858R; 19-del/20-INS; 19-del/T790M; 20-ins; 20-ins/G719X; 22-ins; L861Q; G719X; G719X/L861Q; S768I; L858R; L858R/S768I; L858R/T790M; L858R/20-ins; L859R; L861Q and WT.

In total, *EGFR* and *KRAS* mutations, as well as *EML4-ALK* rearrangements, were detected in 618 patients. Most of these changes were *EGFR* mutations. The *EGFR* mutations identified including exon 19 deletions, exon 20 insertions, G719X, S768I, L858R, etc. (Fig. 2). Exon 19 deletions and L858R were the two most frequent mutations. The mutation frequency of the *EGFR* and *KRAS* genes appeared to be affected by gender (*P* < 0.05). Female patients exhibited a higher rate of *EGFR* mutations [297 of 436 (68.12%) vs. 215 of 597 (36.01%) male patients), whereas male patients had more *KRAS* mutations [40 of 597 (6.70%) vs. 13 of 436 (2.98%) female patients]. Moreover, 20 patients exhibited complex mutations, with a combination of exon 19 deletions and L858R.

### Phenotype-genotype relationships

Nucleotide changes in the *EGFR* gene accounted for most of the mutations detected. A total of 512 patients, most of whom were middle-aged, had various *EGFR* mutations. Most *EGFR* mutation related tumors that were approximately 11–30 mm in diameter were detected in non-tobacco-using patients and in stage I according to the TNM classification; 275 of 297 (92.59%) female patients and 102 of 215 (47.44%) male patients had never smoked. The rest clinicopathological data were not affected by gender in the *EGFR* mutation patients, except for smoking status.

Compared with the wild-type *EGFR* patients, the *EGFR* L858R mutation patients demonstrated clear differences in terms of tumor site, pathological stage and type, tobacco use status, tumor size and visceral pleura invasion status. Most tumors with *EGFR* L858R mutations were located on the right side, whereas tumors with non-*EGFR* mutations were on the left side. Although the age profile appeared to be similar between these two groups, slight differences were observed when the age profiles were analyzed by gender. L858R mutation female patients were older than those with non-*EGFR* mutations.

Tumors with *EGFR* 19 del mutations were also more likely to occur on the right side. This patient group had more advanced tumors (stage III) compared with the *EGFR* L858R mutation and wild-type patients. Invasion of the visceral pleura was also common in this group.

Of the 1033 lung cancer patients, 13 females and 40 males harbored *KRAS* mutations. Most female patients with *KRAS* mutations were between 31 and 60 years old, whereas male patients with *KRAS* mutations were 51 to 70 years old. Left-side cancers were more common in *KRAS* mutation female patients. Most of these tumors were in stage I, and *KRAS* mutation female patients demonstrated a lower percentage of stage II and III disease compared with *EGFR* mutation females (*P* < 0.05). Only 7 (17.50%) of the *KRAS* mutation male patients had never smoked. For females, only 1 patient smoked at the time of diagnosis. The tumor volume of the *KRAS* mutation patients were larger than those of the *EGFR* mutation patients (*P* < 0.05). Furthermore, the tumor size of female patients was greater than that of the male patients.

The *EML4-ALK* rearrangement was observed in 53 patients. Very few differences were observed between female and male patients. For males, the non-tobacco using group exhibited a higher frequency of visceral pleura invasion. Seven (23.33%) of these cases were squamous carcinoma (Table 2).

**Figure 3:**
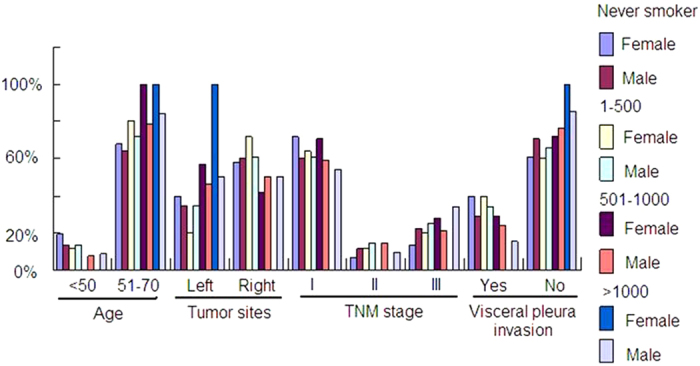
The clinicopathological characteristics of the different smoking status subgroups.

Compare with patients who never smoked, heavy smokers were more likely to be elder and to possess squamous tumors that were larger in size. Most of these tumors were located on the left side and were classified as stage III according to the TNM classification (Fig. 3).

## Discussion

Despite the advances achieved in diagnosis and treatment, the overall prognosis of lung cancer remains poor. The 5-year survival rate remains below the expected rate. Efforts to improve the survival of lung cancer patients are currently focused on the development of innovative treatment options, particularly novel target-based therapies directed against key signaling pathways involved in the development and progression of lung cancer^10^. In the past decades, the application of specifically targeted medication has greatly improved the treatment outcomes of lung cancer patients. TKIs, including gefitinib and erlotinib, have become the standard first-line therapy for patients with advanced NSCLC that harbor activating *EGFR* mutations^11^,^12^, especially for women and patients who have never smoked. This categorization is based on the fairly salient fact that most clinically relevant *EGFR* mutations in lung cancer patients are either deletions in exon 19 or missense mutations in exon 21^13^. In the Iressa Pan-Asia Study (IPASS), patients with *EGFR* mutations exhibited great improvement in the objective response rate and longer progression-free survival after receiving gefitinib compared with patients without *EGFR* mutations^11^. Therefore, the molecular profiling of patients is crucial for specific treatments.

The accurate analysis of clinicopathological and molecular genetic characteristics of target patients will help clinicians select an optimal treatment plan. In this study, we analyzed the clinicopathological features of 1033 Chinese lung cancer patients. The incidence of mutation was significantly higher in women. Similar results have been reported by a previous study, which found a higher incidence of lung cancer in female non-smokers. In our study, the detected *EGFR* gene mutations were also primarily found in patients who had never smoked.

Recently, non-tobacco-using-patients with lung cancers have received considerable attention with the application of EGFR TKIs. These drugs have demonstrated higher responses in specific non-tobacco-using groups: Asian females with adenocarcinoma-type histology and *EGFR* mutations. Among these clinicopathological factors, the most significant factor is the *EGFR* gene mutation. Activating *EGFR* mutations, including exon 19 deletions and a missense mutation (L858R) in exon 21, have been found to be the most powerful biologic predictors of EGFR TKI sensitivity^14^. However, using *EGFR* mutational screening to anticipate responses to EGFR TKI treatment is often impractical for clinicians. Therefore, more meticulous and accurate clinical data that can better predict treatment outcomes are urgently required. Our study summarized the characteristics of Chinese lung cancer patients with *EGFR* mutations. Patients, especially never smoked females, who were 51–70 years old, and had 1–20 mm stage I adenocarcinomas on the right side were more likely to possess *EGFR* mutations. Patients with these characteristics may benefit from EGFR TKI therapy.

Moreover, 53 of our 1033 patients (5.13%) had an *EML4-ALK* translocation, which involves the fusion of the N-terminus of *echinoderm microtubule associated protein-like 4 (EML4*) and the intracellular domain of *ALK*. The 23 female patients demonstrated features that were similar to those of the entire patient group, whereas the 30 male patients exhibited unique profiles. Although these tumors were of a smaller volume [the diameter of 46.67% (14 of 30) of the samples was approximately 1–20 mm], 56.67% (17 of 30) invaded the visceral pleura. Further, a high proportion (16 of 30, 53.33%) of non-smokers with low-grade (21 of 30, 70%) adenocarcinomas was observed. The difference between the *EGFR* mutant and *EML4-ALK* rearrangement patients also explained the clinical observation that tumors with an *EML4-ALK* translocation did not respond positively to EGFR TKI therapy^15^. The future development of a targeted agent is required for this patient subgroup.

The frequency of *KRAS* mutations varies among different ethnic groups, ranging from 19% to 30% in NSCLC^16^. These mutations, which occurred most frequently in codons 12 and 13^17^, were more common in female and younger patients as well as patients with adenocarcinoma. *KRAS* mutations showed close relationship with a history of smoking. However, 6–15% of lung adenocarcinoma patients who had never smoked still harbored a *KRAS* mutation^18^.

In our study, the *KRAS* mutation rate was extremely low in both the entire patient group (53 of 1033, 5.13%) and the adenocarcinoma patient group (45 of 759, 5.93%), which is consistent with a previous conclusion that Asians exhibit a lower frequency of *KRAS* mutations. Compared with the *EGFR* mutation and *EML4-ALK* rearrangement patients, *KRAS* mutation patients demonstrated unique features in terms of tumor site, pathology stage and smoking status. Tumors occurring on the right side and in male smokers over the age of 50 were more likely to harbor *KRAS* mutations. Larger tumor size, stage III classification, and reduced visceral pleura invasion were also more frequently observed. This specific group deserves more attention in the development of novel drugs because these patients were not sensitive to current adjuvant chemotherapy^17^.

In this study, we summarized the characteristics of Chinese lung cancer patients, especially in terms of different driver gene mutation subgroups. The distinct features of the different subgroups could help clinicians select more specific and effective treatments for patients. Furthermore, our results demonstrated the necessity to develop novel therapeutic agents for non-*EGFR* mutation patients. Besides, the small size of the *KRAS* mutation group prevented us from obtaining detailed clinical information. A further study in a large population would be helpful for developing more productive treatments.

## Additional Information

**How to cite this article**: Yang, Y. *et al.* Elderly male smokers with right lung tumors are viable candidates for *KRAS* mutation screening. *Sci. Rep.* **6**, 18566; doi: 10.1038/srep18566 (2016).

## Figures and Tables

**Table 1:** The clinicopathologic characters of 1033 Chinese lung cancer patients.

	Number	Percentage	Number	Percentage
Gender				
Male	597	57.79%		
Female	436	42.21%		
Age	Female		Male	
<30	3	0.69%	1	0.17%
31–40	13	2.98%	9	1.51%
41–50	67	15.37%	59	9.88%
51–60	145	33.26%	183	30.65%
61–70	157	36.01%	242	40.54%
>70	51	11.70%	103	17.25%
Site				
Left	171	39.22%	243	40.70%
Right	252	57.80%	340	56.95%
Bilateral	13	2.98%	14	2.35%
TNM stage				
Stage I	313	71.79%	359	60.13%
Stage II	33	7.57%	81	13.57%
Stage III	65	14.91%	133	22.29%
Stage IV	25	5.73%	24	4.02%
Smoking				
Never smoker	403	92.43%	221	37.02%
<100	2	0.46%	2	0.34%
101–500	23	5.28%	139	23.28%
501–1000	7	1.61%	189	31.66%
>1000	1	0.23%	46	7.71%
Tumor size (mm)				
1–10	78	17.89%	79	13.23%
11–20	138	31.65%	155	25.96%
21–30	122	27.98%	160	26.80%
31–40	60	13.76%	88	14.74%
41–50	13	2.98%	51	8.54%
51–60	18	4.13%	33	5.53%
61–70	2	0.46%	14	2.35%
71–80	1	0.23%	8	1.34%
81–90	1	0.23%	3	0.50%
91–100	2	0.46%	4	0.67%
>100	1	0.23%	2	0.34%
Visceral pleura invasion				
Yes	170	38.99%	165	27.64%
No	266	61.01%	432	72.36%

**Table 2:** The association between clinicopathologic characters and mutation status of *EGFR* and *KRAS* mutation as well as rearrangement of EML4-ALK in 1033 Chinese lung cancer patients.

	*EGFR mutateon*				*EML4-ALK*				*KRAS mutateon*			
Total	512				53				53			
Sex												
Male	215	41.99			30	56.60%			40	75.47%		
Female	297	58.01			23	43.40%			13	24.53%		
Age	F^1^		M^2^		F^1^		M^2^		F^1^		M^2^	
<30	3	1.01%	0	0.00%	0	0.00%	0	0.00%	0	0.00%	0	0.00%
31–40	10	3.37%	3	1.40%	2	8.70%	0	0.00%	0	0.00%	1	2.50%
41–50	38	12.79%	20	9.30%	4	17.39%	7	23.33%	3	23.08%	2	5.00%
51–60	98	33.00%	70	32.56%	10	43.48%	6	20.00%	4	30.77%	15	37.50%
61–70	109	36.70%	91	42.33%	6	26.09%	13	43.33%	5	38.46%	15	37.50%
>70	39	13.13%	31	14.42%	1	4.35%	4	13.33%	1	7.69%	7	17.50%
Site												
Left	115	38.72%	77	35.81%	6	26.09%	8	26.66%	7	53.84%	17	42.50%
Right	173	58.25%	129	60.00%	16	69.57%	21	70.00%	6	46.15%	23	57.50%
Bilateral	9	3.03%	9	4.19%	1	4.35%	1	3.33%	0	0.00%	0	0.00%
TNM stage												
Stage I	212	71.38%	139	64.65%	17	73.91%	22	73.34%	8	61.54%	26	65.00%
Stage II	18	6.06%	12	5.58%	1	4.35%	2	6.66%	4	30.76%	5	12.50%
Stage III	50	16.84%	52	24.91%	5	21.74%	6	20.00%	1	7.69%	9	22.50%
Stage IV	17	5.72%	12	5.58%	1	4.35%	1	3.33%	0	0.00%	3	7.50%
Smoking												
Never smoker	275	92.59%	102	47.44%	22	95.65%	16	53.33%	12	92.31%	7	17.50%
<100	2	0.67%	1	0.47%	0	0.00%	1	3.33%	0	0.00%	0	0.00%
101–500	16	5.39%	53	24.65%	1	4.35%	7	23.33%	1	7.69%	9	22.50%
501–1000	4	1.35%	48	22.33%	0	0.00%	5	16.67%	0	0.00%	20	50.00%
>1000	0	0.00%	11	5.12%	0	0.00%	1	3.33%	0	0.00%	4	10.00%
Tumor size												
1–10	47	15.82%	25	11.63%	4	17.39%	3	10.00%	1	7.69%	4	10.00%
11–20	95	31.99%	65	30.23%	4	17.39%	11	36.67%	3	23.08%	9	22.50%
21–30	97	32.66%	73	33.95%	9	39.13%	7	23.33%	1	7.69%	12	30.00%
31–40	40	13.47%	31	14.42%	3	13.04%	2	6.67%	4	30.77%	3	7.50%
41–50	8	2.69%	7	3.26%	1	4.35%	2	6.67%	0	0.00%	5	12.50%
51–60	8	2.69%	6	2.79%	0	0.00%	3	10.00%	2	15.38%	5	12.50%
61–70	0	0.00%	2	0.93%	2	8.70%	0	0.00%	0	0.00%	2	5.00%
71–80	1	0.34%	4	1.86%	0	0.00%	2	6.67%	0	0.00%	0	0.00%
81–90	0	0.00%	1	0.47%	0	0.00%	0	0.00%	1	7.69%	0	0.00%
91–100	1	0.34%	0	0.00%	0	0.00%	0	0.00%	1	7.69%	0	0.00%
>100	0	0.00%	1	0.47%	0	0.00%	0	0.00%	0	0.00%	0	0.00%
Pathological type												
Lung adenocarcinomas	289	97.31%	190	88.37%	23	100.00%	21	70.00%	13	100.00%	32	80.00%
Squamous carcinoma	0	0.00%	11	5.12%	0	0.00%	7	23.33%	0	0.00%	4	10.00%
Large cell carcinoma	0	0.00%	5	2.33%	0	0.00%	1	3.33%	0	0.00%	1	2.50%
Small cell carcinoma	0	0.00%	3	1.40%	0	0.00%	0	0.00%	0	0.00%	0	0.00%
Other	8	2.69%	6	2.79%	0	0.00%	1	3.33%	0	0.00%	3	7.50%
Visceral pleura Invasion												
Yes	129	43.43%	89	41.40%	11	47.83%	17	56.67%	6	46.15%	13	32.50%
No	168	56.57%	126	58.60%	12	52.17%	13	43.33%	7	53.85%	27	67.50%

1:Female; 2:Male.
